# Induction of the Epithelial-to-Mesenchymal Transition of Human Colorectal Cancer by Human TNF-β (Lymphotoxin) and its Reversal by Resveratrol

**DOI:** 10.3390/nu11030704

**Published:** 2019-03-26

**Authors:** Constanze Buhrmann, Mina Yazdi, Bastian Popper, Ajaikumar B. Kunnumakkara, Bharat B. Aggarwal, Mehdi Shakibaei

**Affiliations:** 1 Musculoskeletal Research Group and Tumor Biology, Chair of Vegetative Anatomy, Institute of Anatomy, Faculty of Medicine, Ludwig-Maximilian-University Munich, Pettenkoferstrasse 11, D-80336 Munich, Germany; constanze.buhrmann@med.uni-muenchen.de (C.B.); Mina.Yazdi@med.uni-muenchen.de (M.Y.); 2 Biomedical Center, Core Facility Animal Models, Ludwig-Maximilian-University Munich, D-82152 Martinsried, Germany; Bastian.Popper@bmc.med.lmu.de; 3 Cancer Biology Laboratory & DBT-AIST International Laboratory for Advanced Biomedicine (DAILAB), Department of Biosciences & Bioengineering, Indian Institute of Technology Guwahati, Assam 781039, India; ajai78@gmail.com; 4 Anti-inflammation Research Institute, San Diego, CA 92126, USA; bbaggarwal@gmail.com

**Keywords:** resveratrol, colorectal cancer, TNF-β (lymphotoxin α), epithelial-to-mesenchymal-transition (EMT), NF-κB

## Abstract

Objective: Tumor necrosis factor-beta (TNF-β), as an inflammatory mediator that has been shown to promote tumorigenesis, induces NF-κB. Natural multi-targeted agent resveratrol in turn shows anti-inflammatory and anti-cancer properties. Epithelial-to-mesenchymal transition (EMT) allows cancer cells to turn into a motile state with invasive capacities and is associated with metastasis and development of cancer stem cells (CSC). However, TNF-β-induced EMT and the anti-invasion mechanism of resveratrol on CRC are not yet completely understood. Methods: We investigated the underlying molecular mechanisms of resveratrol on TNF-β/TNF-βR-induced EMT and migration of CRC cells (HCT116, RKO, SW480) in monolayer or 3D alginate cultures. Results: TNF-β, similar to TNF-α, induced significant cell proliferation, morphological change, from an epithelial to a spindle-like mesenchymal shape with the formation of filopodia and lamellipodia associated with the expression of EMT parameters (elevated vimentin and slug, reduced E-cadherin), increased migration/invasion, and formation of CSC in all CRC cells. Interestingly, these effects were dramatically decreased in the presence of resveratrol or anti-TNF-βR with TNF-β co-treatment, inducing biochemical changes to the mesenchymal-epithelial transition (MET), with a planar cell surface and suppressed formation of CSC cells. This was associated with a significant increase in apoptosis. Furthermore, we found that resveratrol suppressed TNF-β-induced NF-κB and NF-κB-regulated gene biomarkers associated with growth, proliferation, and invasion. Finally, TNF-βR interacts directly with focal adhesion kinase (FAK) and NF-κB. Conclusion: These results suggest that resveratrol down-regulates TNF-β/TNF-βR-induced EMT, at least in part via specific suppression of NF-κΒ and FAK in CRC cells.

## 1. Introduction

In industrialized countries, colorectal cancer is the third most common cancer diagnosed and ranks second in terms of mortality [1]. It is estimated that by the year 2035, mortality rates for colon cancer would have increased by 60% [2]. The main reason for colon cancer-related mortality is resistance to current standard chemotherapeutic treatments, resulting in high incidence of metastatic recurrence [3,4]. This highlights the need for novel therapeutic approaches, which may enhance chemosensitivity and suppress metastatic recurrence and thus improve overall survival rates.

Chronic inflammation presents a critical component of tumor initiation and progression [5]. The nuclear factor “kappa-light-chain-enhancer” of activated B-cells (NF-κB) is an important transcription factor for the immune defense and is involved in regulating cell proliferation and apoptosis [6,7]. In several cancers, NF-κB is constitutively activated, stimulating pro-inflammatory cytokines’ activation and chemokines, promoting proliferation and suppressing apoptosis [8,9]. Hereby, activation of pro-inflammatory mediators in the tumor microenvironment plays a major role in modulating communication between the tumor cells and their environment, eventually promoting invasion and migration of tumor cells [10]. In particular, NF-κB-dependent activation of members of the Tumor-Necrosis-Factor-superfamily has been identified as being critically involved in this process [9]. Indeed Tumor Necrosis Factor-alpha (TNF-α) and its stimulating role in cancer has been well studied [11], but the role of its closest homologue TNF-β (alias Lymphotoxin-α) remains to be elucidated.

We and others have previously shown that TNF-β can, like TNF-α, modulate proliferation, survival, invasion, migration, and colony formation in colorectal and ovarian cancer [12,13]. Indeed, similar to TNF-α, TNF-β stimulates an inflammatory cascade in cancer cells and investigations on ovarian tumors have shown that TNF-β is over-expressed in cancer cells and associated fibroblasts [12]. It has previously been shown that TNF-β may activate canonical and non-canonical NF-κB pathways [14,15,16,17] and signals through the lymphotoxin-β receptor (LTβR) [18]. Interestingly, induction of NF-κB by binding of TNF-β to LTβR has previously been shown to activate cell death in HT29 adenocarcinoma cells [19].

Evidence has shown that inflammation in the tumor microenvironment acts as a potent promoter, converting cells into a motile state by stimulating epithelial-to-mesenchymal-transition (EMT) in cancer cells [20]. The cancer cell’s loss of stationary properties, cell–cell and cell–matrix bound properties, is associated with loss of inter- and extracellular junctions and remodeling of cell–matrix interaction [21]. A major marker for the loss of epithelial properties is E-Cadherin suppression, leading to a loss of adherence and polarized organization of epithelial cells [22,23,24]. Furthermore, EMT is known to promote the formation of cancer stem cells [25,26] and activate drug resistance [27]. Interestingly, the pathways leading to EMT and malignancy of cancer cells are tightly regulated by multiple mediators at the epigenetic and post-translational level [24].

Resveratrol (*trans*-3,5,4’-Trihydroxystilben), a natural polyphenol, has been associated with a variety of different medicinal effects, like anti-inflammatory and anti-neoplastic properties [28,29,30]. In cancer, resveratrol has been shown to up-regulate intercellular junctions and focal adhesion molecules, block EMT, suppress inflammatory pathways, and initiate apoptosis [30,31,32].

We have previously shown that TNF-β-induced CRC cell (HCT116, HCT116R) migration from 3D-alginat cultures and TNF-β has increased chemoresistance in CRC cells to 5-FU. Furthermore, resveratrol could significantly chemosensitize CRC cells to 5-FU in a TNF-β-promoted inflammatory tumor microenvironment [13]. Very recently, we have reported that a TNF-β signaling pathway is involved in proliferation in CRC cells and that resveratrol down-regulates the TNF-β-mediated inflammatory response by suppressing NF-κB promotion, which regulates the proliferation of tumor cells [33]. In addition, it has been reported that the EMT is a crucial mechanism of cancer progression [25,26], however, whether TNF-β induces migration and EMT in various colorectal cancer cells has not yet been evaluated and needs to be further investigated. Therefore, we performed a comparative morphological, ultrastructural, and biochemical study to examine the modulatory effect of resveratrol on EMT and migration caused by TNF-β/TNF-β-receptor signaling, using three different CRC cell lines. In the present report, we provide evidence that resveratrol suppresses the TNF-β signaling pathway via the TNF-βR to decrease EMT, further highlighting its significant implications for inflammation-induced tumor promotion, particularly in CRC.

## 2. Materials and Methods

### 2.1. Antibodies, Cytokines, and Chemicals

Anti-CXCR4 (CXC-Motiv-Chemokinreceptor 4) was acquired from Abcam PLC (Cambridge, UK). Anti-CD133, -ALDH1, and -CD44 were purchased from Antibodies-online. Anti-phospho-specific p65 (NF-κB) and anti-MMP-9 were from R&D Systems (Heidelberg, Germany). Anti-E-cadherin, anti-vimentin, and anti-slug were obtained from Santa Cruz Biotechnology (Santa Cruz, CA, USA). Anti-FAK was purchased from Becton Dickinson (Heidelberg, Germany). Monoclonal antibodies to Ki-67 and secondary antibodies used for fluorescence labelling were purchased from Dianova (Hamburg, Germany). Anti-β-actin, alginate, resveratrol, and *Staphylococcus aureus* bacteria were from Sigma–Aldrich Chemie (Munich, Germany). TNF-β, TNF-α, anti-TNF-β-receptor, and anti-TNF-β were purchased from eBiosciences (Frankfurt, Germany). In addition, TNF-β and TNF-α, were kindly provided by Genetech, Inc. (South San Francisco, CA, USA) [34]. Secondary antibodies for Western blotting were from Millipore (Schwalbach, Germany) and gold particle-conjugated secondary antibodies were from Amersham (Braunschweig, Germany). Resveratrol was dissolved in ethanol as stock and stored at –20 °C. Epon was from Plano (Marburg, Germany).

### 2.2. Cell Lines

The human CRC cell lines (HCT116, SW480) used in this study, were from the European Collection of Cell Cultures (Salisbury, UK). The RKO cell line was from the American Type Culture Collection (ATCC). A whole cell culture growth medium, supplemented with 10% FCS, was prepared and cells cultured as previously described in detail [30].

### 2.3. Experimental Design

Human CRC cell (HCT116, SW480, and RKO) monolayer cultures were washed three times with a serum-starved medium (3% FCS) and incubated for 1 h with the same medium. CRC cells were either left untreated or treated with 10 ng/mL of TNF-β or TNF-α alone for 12 h, or pretreated with 5 µM resveratrol by itself or 5 µM resveratrol for 4 h, followed by co-treatment with 10 ng/mL of TNF-β or TNF-α and 5 µM resveratrol for 12 h.

For the invasion assay, serum-starved HCT116, RKO, and SW480 CRC cells in alginate bead culture were left untreated, treated with 5ng/ml or 10ng/mL TNF-β or TNF-α, 5 µM resveratrol, or the combination of resveratrol and TNF-β or TNF-α for 14 days. In an additional approach, HCT116, RKO, and SW480 cells were left untreated, treated with TNF-β, resveratrol, TNF-β and resveratrol, or treated in suspension with an anti-TNF-β-receptor (0.1, 1, 10, 20 µg/mL) for 15 min, and then transferred to the alginate, followed by co-treatment with or without 10 ng/mL of TNF-β or resveratrol for 14 days. These investigations were performed in triplicate and the findings are presented as mean values from three independent investigations.

### 2.4. Immunofluorescence Investigations

CRC cells (HCT116, RKO, and SW480) in monolayer cultures were treated as described above and were subjected to immunofluorescent labelling for slug, vimentin, and E-cadherin as previously described in detail [30]. In a second approach, untreated CRC cells were investigated for TNF-β and TNF-βR immunofluorescence labeling. Briefly, after blocking with 1% BSA/PBS, cells were incubated with primary antibodies, diluted 1:80 in 1% BSA/PBS overnight at 4 °C, washed with PBS, and incubated with secondary antibodies diluted at 1:100 for 1.5 h. Finally, cells were counterstained with a DAPI (4, 6-Diamidino-2-phenylindole, Sigma, Munich, Germany) nuclear stain and visualized through a fluorescent microscope (Leica, Germany). All investigations were performed at least in triplicate and the percentage of positively labelled cells was quantified by counting 600–800 cells in 10 microscopic fields.

### 2.5. Three Dimensional Alginate Culture

Cultivation of CRC cells in three-dimensional in vitro culture was performed as alginate bead culture, as previously described [30,32,35]. The alginate tumor microenvironment culture provides an in vivo close environment and is extremely suitable for studying early events in tumorigenesis.

### 2.6. Invasion and Colony Forming Assay

The influence of resveratrol and/or TNF-β/TNF-α on invasion and colony formation capacity of CRC cells was investigated in alginate bead culture, as previously described [30,35]. Cells that had migrated through the alginate matrix and formed newly adhered colonies on the bottom of the petri dishes were labeled with toluidine blue and quantified by counting all colonies under a microscope. Every investigation was repeated in triplicate, data were compared to the control, and statistically significant values with *p* < 0.05 are shown by one asterisk (*); *p* < 0.01 by two asterisks (**).

### 2.7. Cell Viability Investigation from Alginate Culture

Cell viability of CRC cells in alginate bead culture was assessed by applying the MTT method as previously stated [32]. Briefly, serum-starved CRC cells were left untreated or treated, as described above, and then cultivated for 14 days in alginate cultures. To retrieve CRC cells from the alginate, beads were dissolved in 55 mM sodium citrate solution for 20 min, as described previously [36]. Finally, viability of CRC cells was determined using a revelation 96-well multiscanner plate ELISA reader (Bio-Rad Laboratories Inc. Munich, Germany), by measuring the Optical Density at 550 nm (OD550). The investigation was performed in triplicate and the data are provided as mean values with standard deviations from three independent experiments.

### 2.8. Ultrastructural Investigations

Ultrastructural investigations of monolayer cultures were performed to evaluate the effect of resveratrol and/or TNF-β on CRC cells, as previously stated [35]. CRC monolayer cultures were fixed for 1 h in Karnovsky fixative, followed by post-fixation in 1% O_s_O_4_ solution. After dehydration in an ascending alcohol series, cells were embedded in Epon and cut ultrathin with a Reichert-Jung Ultracut E (Darmstadt, Germany). Ultrathin sections were contrasted with uranyl acetate/lead citrate and evaluated with a transmission electron microscope (Jena, Germany) or a Jeol 1200 EXII (Akishima Tokyo, Japan). For statistical evaluation of apoptotic cells, 100 cells from 25 microscopic fields were counted manually and all obtained data were compared to the control and statistically significant differences were highlighted with *p* < 0.05 (*); *p* < 0.01 (**).

### 2.9. Pre-Embedding for Ultrastructural Immunoelectron Microscopy

Monolayer cultures of CRC cells were left untreated or treated, as stated above. The effect of resveratrol and/or TNF-β on EMT marker E-Cadherin and vimentin in CRC cells was investigated by an ultrastructural immunoelectron pre-embedding technique, as previously described [32]. Briefly, cells in suspension were incubated with a primary antibody for 15 min and diluted 1:50 in a cell culture medium at an ambient temperature (AT). After washing with PBS three times, cells were incubated with 10 nm goat anti-rabbit gold-conjugated secondary antibodies (diluted 1:50 in cell culture medium) for an additional 10 min at AT. After fixation with 2% glutaraldehyde for 5 min, followed by 1% O_s_O_4_ solution for 5 min, cells were dehydrated in a series of ethanol concentrations and finally embedded in Epon. Ultrathin sections were prepared and examined using a Zeiss transmission electron microscope (Jena, Germany) or a Jeol 1200 EXII (Akishima Tokyo, Japan).

### 2.10. Immunoblotting Investigations

HCT116, RKO, and SW480 CRC cells in alginate bead culture were left untreated or treated, as described above. Cell lysates for immunoblotting investigations were extracted from CRC cells in alginate bead cultures, as previously described [30,35]. The investigations were performed in triplicate, and β-actin was applied as a control and to normalize the protein quantities.

### 2.11. Immunoprecipitation Assay

Immunoprecipitation investigations were performed to elucidate the relationship between TNF-β-receptor and endogenous signaling interactions with focal adhesion kinase (FAK) or with NF-κB, as previously described [37,38]. CRC cells were either left untreated or treated with TNF-β (10 ng/mL) in suspension for 10 min and then transferred to the alginate for 3 days. For immunoprecipitation, cell extracts were incubated with 25 µl rabbit and mouse IgG serum and *Staphylococcus aureus* bacteria to pre-clear samples. They were incubated with antibodies against TNF-β-receptor in a wash buffer (0.1% Tween 20, 150 mM NaCl, 50 mM Tris-HCl, pH 7.2, 1 mM CaCl_2_, 1 mM MgCl_2_, and 1 mM PMSF) for 2 h at 4 °C, followed by 30 min incubation with *Staphylococcus aureus* bacteria. Finally, obtained immune complexes were washed with a lysis buffer. Samples were reduced with 2-mercaptoethanol, subjected to SDS-PAGE under reducing conditions, and were blotted onto a nitrocellulose membrane, using a transblot apparatus (Bio-Rad, Munich, Germany).

### 2.12. Statistical Analyses

The data were expressed as the mean ± SE and statistical significance between the groups was compared by one-way, two-way, or a three-way ANOVA, and the Wilcoxon–Mann–Whitney test. Each investigation was performed at least three times. A *p*-value < 0.05 was considered statistically significant.

## 3. Results

The goal of this paper was to investigate the effect of resveratrol on TNF-β-induced tumorigenesis of human colorectal cancer cells (CRC) through regulation of EMT. We used three well-described CRC cell lines (HCT116, RKO, and SW480) in a monolayer, as well as a 3D alginate tumor microenvironment culture to answer this question. The amount of ethanol or DMSO used in our study had no suppressive effect on the viability of cells.

### 3.1. TNF-β Induces EMT in CRC Cells and Resveratrol Reverses That

**Figure 1:**
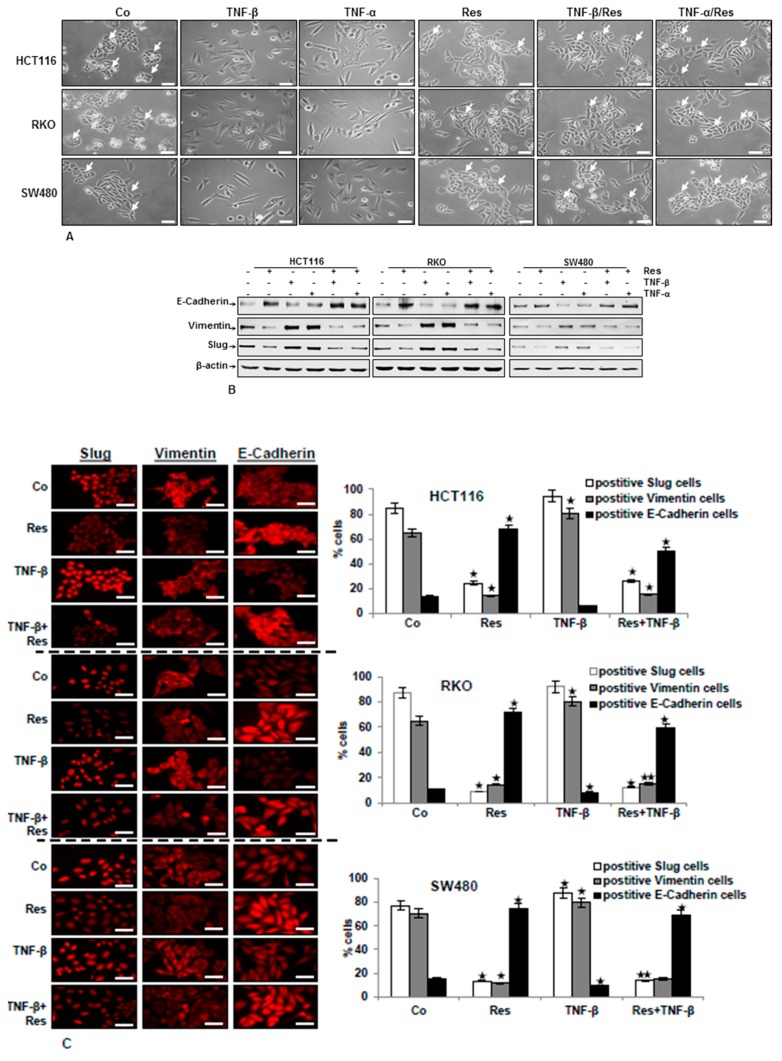
Impact of resveratrol on TNF-β-induced epithelial-to-mesenchymal transition (EMT) and EMT-related proteins in CRC cells (HCT116, RKO, SW480). CRC cells in monolayer culture were either left untreated (Co) or treated with 5µM resveratrol (Res), 10 ng/mL TNF-β, or 10ng/mL TNF-α for 12 h, or cells were pre-treated with resveratrol (5µM) for 4 h, followed by co-treatment with 10ng/mL TNF-β or 10ng/mL TNF-α for 12 h. (**A**) Cell phenotype changes associated with EMT, as demonstrated in phase contrast images. Cell colonies (arrows). Scale bar = 100 μm. (**B**) Immunoblotting of cell lysates was performed for E-cadherin, vimentin, and slug. The housekeeping protein β-actin served as an internal loading control. (**C**) Immunolabeling was done with primary antibodies for E-cadherin, vimentin, and slug, followed by incubation with rhodamine-coupled secondary antibodies. All experiments were performed at least in triplicate and quantification of positively labelled cells was done by counting 600–800 cells from 10 microscopic fields. All data were compared to the control and statistically significant values with *p* < 0.05 were designated by (*) and *p* < 0.01 were designed by (**). Original magnification, 600×. Scale bar = 30 nm.

Since EMT plays an important role in tumor invasion and metastasis [39], we first investigated whether TNF-β with or without resveratrol leads CRC cells to EMT cell morphology by light microscopy. HCT116, RKO, and SW480 cells in monolayer cultures were treated as delineated in Materials and Methods. As indicated in Figure 1, in all three CRC cell lines, control cultures and monolayer cultures treated with resveratrol showed typical colonies (arrows) with an overall epithelial cell shape and tight cell-to-cell contacts (Figure 1A). Interestingly, treatment with resveratrol significantly enhanced cell-to-cell contact, and the majority of cells formed a more rounded cell shape (Figure 1A). However, after treatment with 10ng/mL TNF-β or TNF-α for 12 h, all three CRC cells changed their shape from a typical epithelial to a spindle-like fibroblast-like appearance. The cell-to-cell contacts became detached and cells showed a more mesenchymal shape with less colony origin (Figure 1A). By contrast, in all cell lines, co-treatment with resveratrol and TNFs led markedly to an epithelial cell cluster with increased cell-to-cell contacts (Figure 1A).

To confirm the morphological results described above and to show more precisely the identity of the epithelial-to-mesenchymal-transition (EMT) biomarker, whole cell extracts were probed for slug, vimentin, and E-cadherin expression. Western blotting analysis showed that in CRC cells, TNF-β, similar to TNF-α, reduced the epithelial marker (E-cadherin) and elevated the mesenchymal markers (vimentin) and EMT-related transcription factor (slug) on the protein level, and resveratrol alone or in co-treatment with TNFs significantly decreased vimentin and slug levels and increased E-cadherin expression (Figure 1B).

Furthermore, to confirm the morphological and western blotting results that the CRC cells underwent EMT by TNF-β treatment, which is inhibited by resveratrol, we investigated the expression of EMT markers and the EMT-related transcription factor by immunofluorescence analysis. The untreated control cells demonstrated a minimum amount of E-cadherin, vimentin, and slug expression. On the other hand, the expression of vimentin and slug was markedly elevated, and E-cadherin was reduced in the presence of TNF-β alone (Figure 1C). Treatment of CRC cells with resveratrol alone or in combination with TNF-β clearly demonstrated the inhibition of vimentin, the transcription factor slug, and increasing of E-cadherin expression (Figure 1C). Overall, these results demonstrate that all three CRC cell lines underwent EMT after TNF-β-treatment and that resveratrol can inhibit this induction, inducing biochemical and functional changes towards to MET. Furthermore, the suppression of EMT by resveratrol was not cell type specific, because EMT was inhibited in HCT116, RKO, and SW480 cells.

### 3.2. Resveratrol Suppresses CRC Cell Malignancy by Modifying the Ultrastructure of the Cell Surface Protein

**Figure 2:**
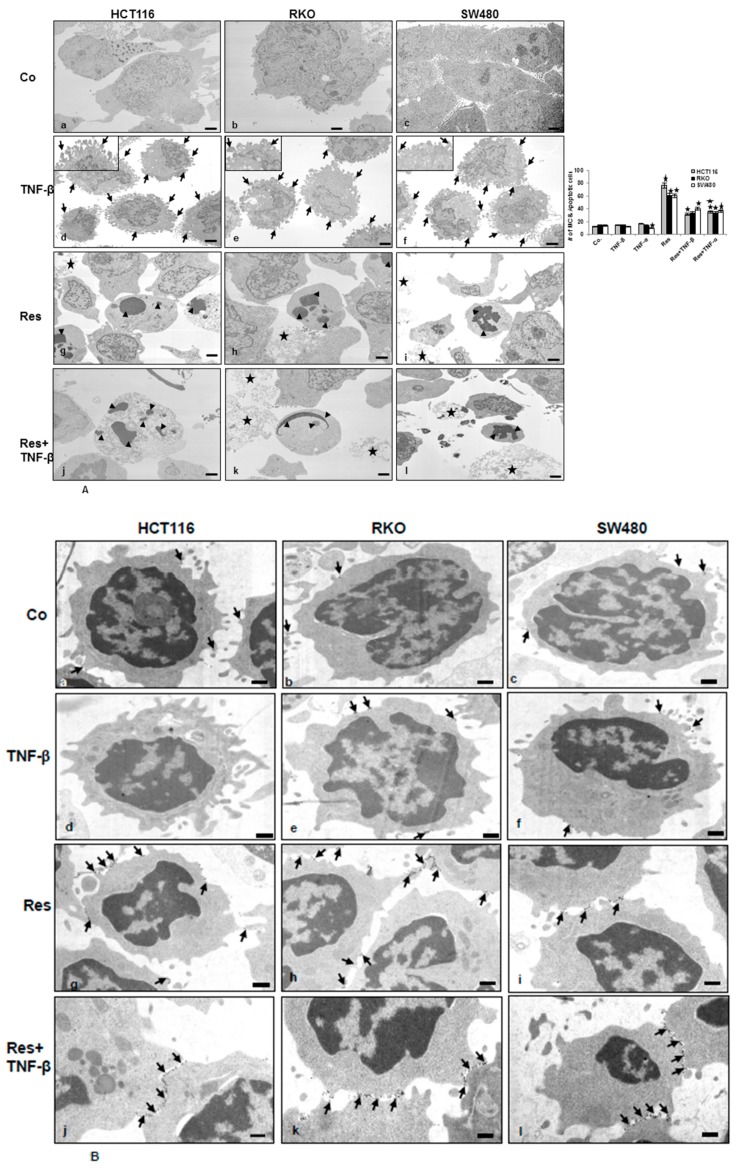
Electron microscopic observation of cell-surface and cell-morphology in CRC cells after treatment with TNF-β or/and resveratrol. HCT116, RKO, and SW480 were cultured in a monolayer culture and were untreated (**a–c**), or treated with TNF-β (**d–f**) or TNF-α with resveratrol (**g–i**) alone, or with TNF-β and co-treated with resveratrol (**j–l**), or TNF-α and co-treated with resveratrol, as described above and investigated with a transmission electron microscope. (**A**) Untreated control (Co) tumor cells showed a planar surface and moderate microvilli (arrows) on the cell surface (a–c). By contrast, treatment with TNF-β or TNF-α resulted in alteration of cell surface ultrastructure and cells contained abundant, long microvilli and filopodia-like structures on the cell surface (d–f). By contrast, treatment of tumor cells with resveratrol alone (g–i) or co-treated cells with resveratrol and TNF-β (j-l) displayed only a few microvilli, with an overall planar surface. Tumor cells became rounded and the nucleus contained more heterochromatin, multiple vacuoles, and swelling of cell organelles like mitochondria. Apoptotic bodies (arrowheads) and cell lysis (*) was visible. a–l: Magnification, 5000x. Scale bar = 1 μm. Insets: Scale bar = 0.09 µm. To quantify mitochondrial changes (MC) and apoptosis in tumor cells, 100 cells from 25 microscopic fields were counted. Values were compared with the control and statistically significant values with *p* < 0.05 were designated by (*) and *p* < 0.01 were designated by (**). (**B**) Effect of resveratrol on E-cadherin expression in CRC cells shown by immunoelectron microscopy. Immunogold labeling (arrows) is observed at the plasma membrane of CRC cells with antibodies against E-cadherin. Scale bars = 0.2 µm.

It is known that EMT is the first step in increasing cancer malignancy and the cell membrane ultrastructure plays a crucial role in cell adhesion and invasion [22,23,24]. To further evaluate the impacts of TNF-β with or without resveratrol on EMT in CRC cells, we examined the alteration on the cell surface ultrastructure by transmission electron microscopy. As shown in Figure 2A, a–c, untreated control cells showed epithelial morphological capacities of cell proliferation and aggregation. Cells were more round to oval and grew in close clusters, with several small microvilli and cell surface processes, a well-developed rough endoplasmic reticulum, Golgi apparatus, and mitochondria, which indicate the maintenance of metabolic active cells (Figure 2A, a–c). Furthermore, CRC cells treated with TNF-β, like TNF-α, clearly exhibited alteration on the cell surface structures associated with increased length of the membrane extensions, microvilli, and long filopodia, reminiscent of mesenchymal-like morphology (Figure 2A, d–f). In the presence of resveratrol alone, the CRC cells revealed an epithelial morphology, planar surface, and, additionally, degenerative changes in cell morphology. Repeated vacuoles appeared, as well as swollen and degenerated cell organelles and cell nuclei containing more condensed heterochromatin and this caused almost complete cell degradation and apoptosis (Figure 2A, g–i). Resveratrol by itself or co-treated with TNF-β, like TNF-α, caused a transformation to an epithelial shape. Cells showed an epithelial cell surface with almost a planar surface and apoptosis (Figure 2A, j–l). Quantification of apoptotic cells supported these findings. Overall, these findings suggest that TNF-β promotes cell morphology alteration and significant spreading on the cell surface, whereas the resveratrol signaling pathway induces an MET phenotype and apoptosis, and slows down cell spreading in all three CRC cells.

We next examined the epithelial morphology induced through resveratrol and cell surface proteins of the MET marker (E-cadherin) in all CRC cells by immunoelectron microscopy. HCT116, SW480, and RKO cells were either untreated or treated, as described before, in a monolayer culture. As demonstrated in Figure 2B, a–c, untreated control cells showed low amounts of the epithelial protein (E-cadherin) (Figure 2B, a–c) diffusely distributed on the round HCT116, SW480, and RKO cells. Furthermore, the TNF-β-treated cells showed diminished E-cadherin expression (Figure 2B, d–f). By contrast, resveratrol induced a round shape with a substantially high expression of E-cadherin (Figure 2B, g–i) and this was seen predominantly on the planar cell body between the cells in CRC cell lines (Figure 2B, g–i). Treatment of CRC cells with resveratrol and/or co-treated with TNF-β showed overexpression and increased levels of E-cadherin (Figure 2B, j–l). Taken together, these findings supported the results in Figure 1 and Figure 2A, that resveratrol induces expression of the adhesion epithelial marker (E-cadherin) in spite of TNF-β treatment in all colon cancer cell lines stimulating biochemical and functional alterations towards MET.

### 3.3. Resveratrol Suppresses TNF-β-Promoted Migration, Colon Cancer Stem Cell Markers, NF-κΒ Activation, and NF-κB-Regulated Gene Products Involved in Proliferation and Metastasis

**Figure 3:**
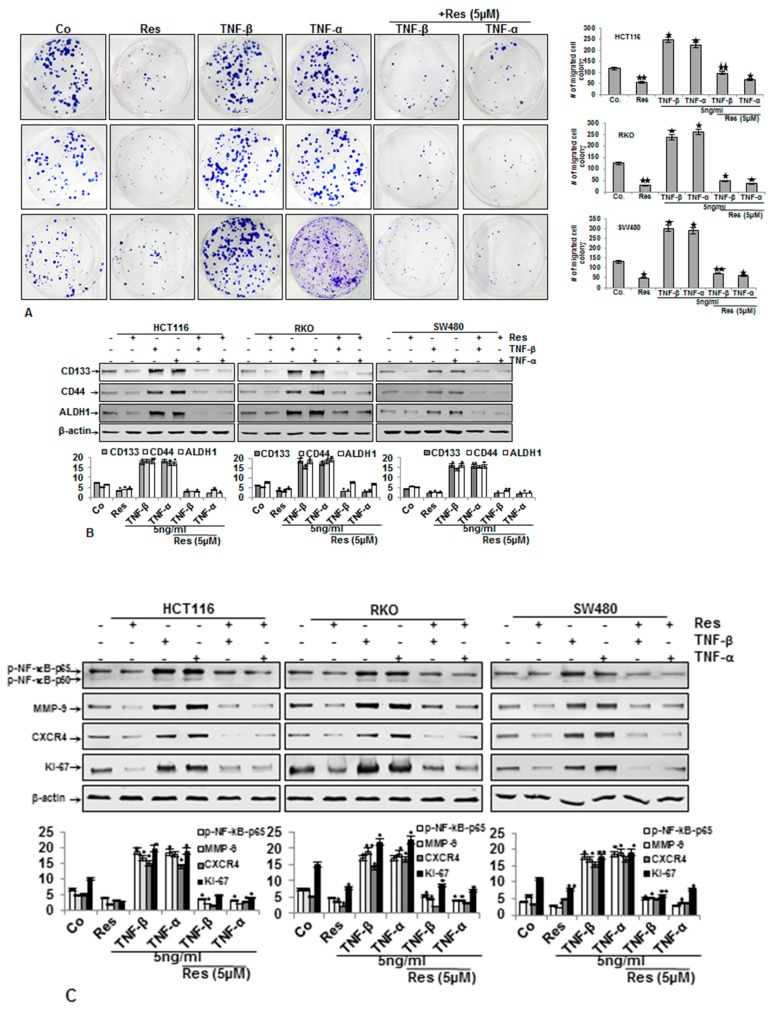
Impact of resveratrol and/or TNF-β on cancer stem cell (CSC) migration, expression of CSC marker, NF-κB, and NF-κB-related proteins involved in proliferation and metastasis. CRC cells were incubated in alginate culture and treated as described in Materials and Methods. (**A**) Migrated colonies were labelled with toluidine blue and the number of migrated colonospheres was counted after 14 days in culture. Experimental data were compared with the control and statistically significant values with *p* < 0.05 were designated (*) and *p* < 0.01 were designated (**). (**B**) Whole cell lysates of alginate beads were made, and immunoblotting was done with antibodies against CD133, CD44, and ALDH1. Results of immunoblot analysis in three different CRC cell lines presented are representative of three individual experiments. The housekeeping protein β-actin was used as a positive loading control. Densitometric analysis of protein expression, as shown with immunoblotting, was done in triplicate. Values were compared with the control and statistically significant values with *p* < 0.05 were shown as (*) and *p* < 0.01 were shown as (**). (**C**). Whole cell lysates of alginate beads were fractionated and immunoblotted with anti-p-NF-κB, anti-MMP-9, anti-CXCR4, anti-Ki-67, and anti-β-actin. Densitometric assessment was done for phospho-p65, MMP-9, CXCR4, and Ki-67. * *p* < 0.05, ** *p* < 0.01. β-actin was used as a loading control in all experiments.

To examine whether resveratrol has a modulatory effect on CRC cell invasion and migration promoted by TNF-β, alginate invasion tests were performed. As shown in Figure 3A, the three CRC cell lines showed significantly amplified cell migration with TNF-β treatment, similar to TNF-α, and resveratrol suppressed this clearly, compared with the corresponding control. Quantification of invasive and adhered colonies confirmed these findings.

Since it was demonstrated that TNF-β, as cytokine, induced the formation of cancer stem cells (CSCs) in HCT116 cells [13], we further investigated whether TNF-β was able to stimulate the formation of CSCs in HCT116, SW480, and RKO cells like TNF-α in 3D alginate cultures and whether resveratrol could modulate the formation of these CSCs. The HCT116, SW480, and RKO cells were cultured in an alginate-beads microenvironment and then treated as described in Materials and Methods. In the alginate, cells formed colonospheres, and we tested the expression of CD133, CD44, and ALDH1 by immunoblotting analysis (Figure 3B). As shown in Figure 3B, consistent with the migration assay findings, CD133, CD44, and ALDH1 protein expression in tumor control culture cells was moderate (Figure 3B). Contrary to that, TNF-β, like TNF-α, clearly stimulated the expression of CSC markers (CD133, CD44, and ALDH1) in all CRC cell lines. Furthermore, resveratrol by itself and/or with TNF-β and/or with TNF-α promoted marked down-modulation of CD133, CD44, and ALDH1 in all CRC cells, compared to the control (Figure 3B). Overall, these findings suggest that TNF-β, like TNF-α, promotes CRC cell activation, progression, and metastasis, increasing CSCs and thereby enhancing the malignancy of the cancer cells. Resveratrol suppresses this, explaining the noticeable targeting of resveratrol for CSCs. Densitometric analysis of immunoblot experiments shows down-regulation of CSC biomarkers in all three CRC cells treated with either TNF-β or TNF-α and/or resveratrol, demonstrating that it is one of the multitargeting cellular and principle mechanisms of resveratrol for tumor suppression (Figure 3B).

A large body of literature indicates that NF-κΒ is a crucial transcriptional mediator of pro-inflammatory cytokines that promote proliferation, invasion, malignancy, and EMT of cancer cells [40,41,42,43]. To further elucidate the underlying pathway, how resveratrol, a multitargeting agent, acts on TNF-β-induced tumorigenesis in CRC cells, we investigated NF-κB phosphorylation and activation of NF-κB-regulated gene products, which are involved in proliferation, invasion, and metastasis. The alginate cultures of all CRC cells were either untreated or treated, as indicated in Materials and Methods. As demonstrated in Figure 3C, the results of immunoblotting showed that resveratrol down-regulated TNF-β-activation of NF-κB and NF-κB-promoted gene proteins MMP-9, Ki-67, and CXCR4, all factors relevant for promoting tumorigenesis, and these were confirmed by quantitative densitometry. Taken together, these findings highlight that specific inhibition of TNF-β-induced NF-κB signaling pathways by resveratrol, at least partially, is one of the crucial anti-tumorigenic mechanisms of resveratrol during tumorigenesis.

### 3.4. Endogenous Demonstration of TNF-β and of TNF-βR on the CRC Cell Membrane

**Figure 4:**
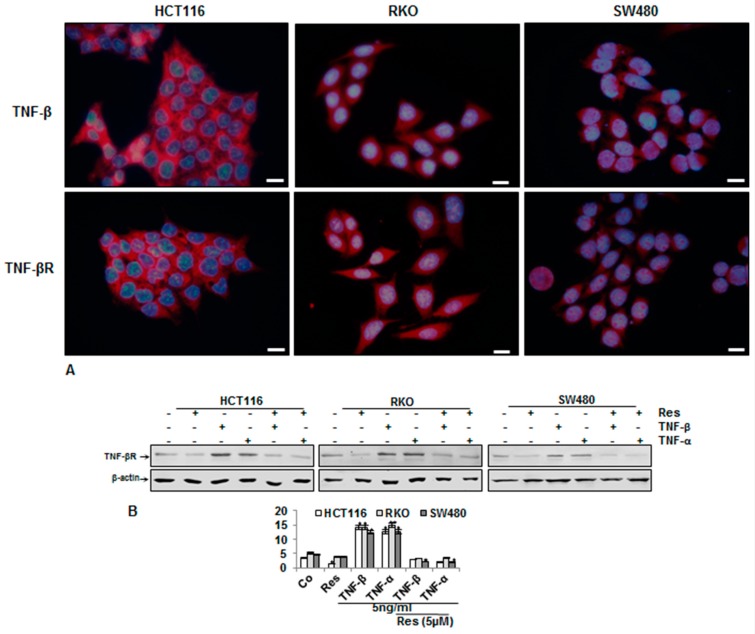
Expression of TNF-β receptor in three CRC cell lines and resveratrol suppressing their expression. (**A**) CRC cells in monolayer cultures were immunolabeled with primary antibodies for TNF-β and TNF-β receptor (TNF-βR), followed by incubation with rhodamine-coupled secondary antibodies and counterstaining with DAPI to visualize cell nuclei. Original magnification, 400x. Scale bar = 30 nm. (**B**) CRC cell lines in monolayer cultures were untreated (Co) or treated, as described above. Whole cell lysates were fractionated and immunoblotted with antibodies against TNF-βR and β-actin. β-actin was used as a loading control in all experiments.

We found that TNF-β was a potent cytokine for promoting the malignancy of cancer cells. To further validate these findings, we also investigated the role of TNF-βR on the migration of all CRC cells. We then investigated the endogenous expression of TNF-β and TNF-βR in different CRC cell lines by immunofluorescence labeling and immunoblotting analysis. Interestingly, in control cultures, immunofluorescence labeling demonstrated that all three CRC cell lines had a basal expression of TNF-β and of TNF-βR protein (Figure 4A). Overall, these results demonstrate that TNF-βR expression is associated with a specific receptor for TNF-β-induced malignancy in cancer cells. More interestingly, we have shown that TNF-β, like TNF-α, activated the expression of TNF-βR, and that resveratrol markedly inhibited either TNF-β- or TNF-α-promoted TNF-βR proteins (Figure 4B). Taken together, these data demonstrate that specific inhibition of TNF-β-promoted TNF-βR pathways in CRC cells by resveratrol, at least partially, is one of the multitargeting mechanisms of anti-tumorigenic properties of resveratrol.

### 3.5. Anti-TNF-βR or Resveratrol Down-Modulates TNF-β-Promoted Proliferation, Invasion, and Colony Formation

**Figure 5:**
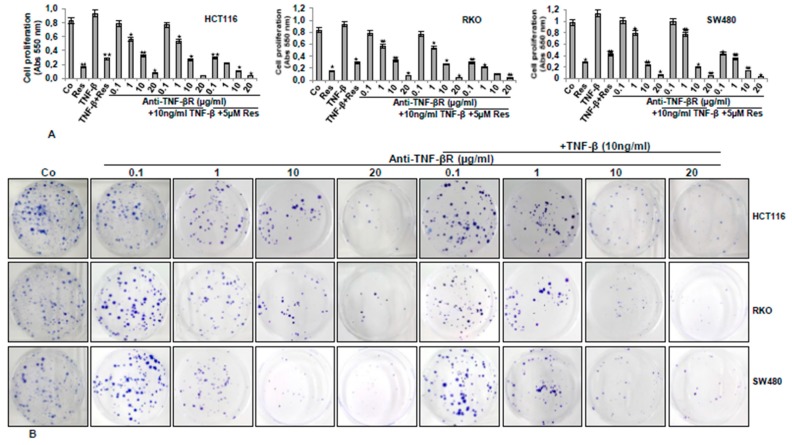
Resveratrol or anti-TNF-βR blocks TNF-β-induced proliferation, migration, and colony formation of CRC cell lines. (**A**) Suppression of the proliferation of CRC cells by resveratrol or anti-TNF-βR, as shown by MTT. CRC cells in alginate culture were untreated (Co) or treated, as described in Materials and Methods. The results were provided by at least three individual experiments. Values were compared with the control, and statistically significant values with *p* < 0.05 were designated by an asterisk (*) and *p* < 0.01 were designated by two asterisks (**). (**B**) Suppression of CRC cell migration and motility by anti-TNF-βR. The migration-based cell motility alginate culture was used to define the relative cell motility of the three CRC cell lines, as described above. After 14 days, migrated cells developed colonies on the bottom of petri dishes and they were fixed and labelled with toluidine blue. Typical alginate culture dishes indicated reduced colony formation by anti-TNF-βR dose-dependently, with or without TNF-β.

Results from Figure 2 and Figure 3 suggest the potential of TNF-β for enhancing survival and metastasis of CRC cells and that resveratrol down-modulates that. Next, we wanted to know whether anti-TNF-βR plays a role in the treatment of CRC alone and/or with TNF-β, and what specific mechanisms participate. HCT116, SW480, and RKO (Figure 5A) cells in alginate were either untreated or treated, as indicated in Materials and Methods, and an MTT assay was performed. The results indicate that TNF-β stimulated CRC cell viability and proliferation, which was significantly inhibited by resveratrol. Interestingly, TNF-βR suppressed cell proliferation in the presence or absence of TNF-β in a dose-dependent manner. In addition, marked inhibition in proliferation of CRC cell lines was shown in an anti-TNF-βR and resveratrol combinational treatment (Figure 5A). We probed further the cell invasion ability of three CRC cell lines, using alginate invasion assay. CRC cell lines in the alginate matrix were treated as indicated in Materials and Methods for 14 days and a migration assay was performed. All CRC cell lines have shown high invasion and colony formation levels, while CRC cell invasion and colony formation was markedly suppressed by anti-TNF-βR treatment dose-dependently, compared to controls (Figure 5B). Taken together, these results indicate clearly, for the first time, that TNF-βR, at least in part, is one of the essential signaling molecules on tumor cells in cytokine (TNF-β)-promoting tumorigenic effects in CRC cells.

### 3.6. TNF-β-Promoted TNF-βR Expression Interacts with FAK and NF-κB in CRC Cells

**Figure 6:**
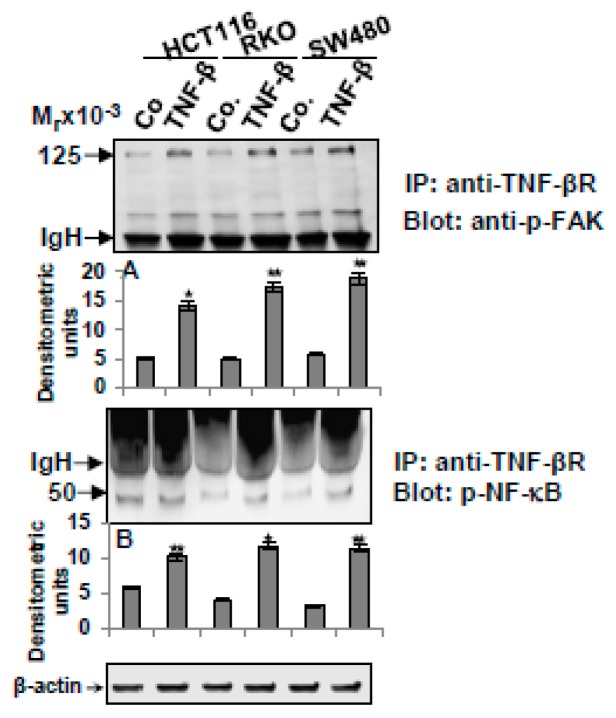
Association of TNF-βR proteins with focal adhesion kinase (FAK) or NF-κB in CRC cells. CRC cells were untreated (Co) or treated with TNF-β (10ng/mL) in suspension for 10 min, and then transferred to the alginate beads for three days. Whole cell lysates were immunoprecipitated (IP) with anti-TNF-βR. Finally, the immunoprecipitates were separated by SDS-PAGE and analyzed by immunoblotting (IB), using anti-p-FAK (**A**) and anti-p-NF-κB (**B**). Densitometric assessment of protein expression, as shown by immunoblotting, was done in triplicate. The original extracts were tested with an antibody to β-actin, as a loading control. Data were compared with the control and statistically significant data with *p* < 0.05 were designated an asterisk (*) and *p* < 0.01 were designated two asterisks (**). IgH = immunoglobulin heavy chain.

We further investigated the metastasis effect of TNF-β and TNF-βR on proteins that contribute to cancer metastasis. Cytoskeleton remodeling, cell adhesion, chemotaxis and focal adhesion kinase (FAK)-signaling pathways, and NF-κB were associated with regulating tumor cell survival, invasiveness, and metastasis [42,43,44,45,46]. We next executed co-immunoprecipitation assays to determine the possible physical interplay of TNF-βR and FAK and/or NF-κB in CRC cell lines in alginate cultures. All three CRC tumor cells were either untreated or treated with TNF-β (10ng/mL) in suspension for 10 min, and then transferred to alginate for three days. Cell lysates were immunoprecipitated with antibodies against TNF-βR, followed by immunoblotting with p-FAK or with p-NF-κB antibodies. As shown in Figure 6, we found in all CRC cells in TNF-β-treated cultures an obvious strong co-immunoprecipitation of the TNF-βR protein with the p-FAK, or with the p-NF-κB protein (Figure 6). In untreated control cultures, marginal TNF-βR co-immunoprecipitation with the p-FAK or p-NF-κB protein was observed (Figure 6). Collectively, these results further suggest TNF-β-promoted TNF-βR-FAK/-NF-κB complex formation during tumorigenesis and it may be one of the important downstream pathways for TNF-β/TNF-βR, leading to malignancy and metastasis in CRC cells.

## 4. Discussion

Previous reports suggested that inflammatory cytokines produced in the cancer microenvironment, such as TNF-α, act as a major promotor and modulator of tumorigenesis through activation of EMT and different signaling pathways, including NF-κΒ [47,48,49,50]. However, the role of the pro-inflammatory cytokine TNF-β during tumorigenesis is still poorly understood and requires further investigation. Moreover, natural compounds as multitargeted agents have the ability to inhibit cancer cell proliferation, survival, EMT, invasion by regulating focal adhesive molecules, cell surface proteins, apoptosis-associated genes, and signal transduction pathways [51,52,53]. Our group has shown previously that resveratrol induces apoptosis and down-modulates focal adhesion kinase (FAK) and inflammatory transcription factors, such as NF-κB, and NF-κB-promoted gene protein expression in CRC cells [13,30,54]. The goal of this investigation was to determine whether TNF-β as a cytokine could serve as a promoter for migration and EMT in various CRC cells and to determine the down-modulating impact of resveratrol on invasion and EMT induced by TNF-β.

In this paper, we showed that TNF-β, similar to TNF-α, stimulated distinctive morphological and molecular changes from epithelial to fibroblast-like mesenchymal phenotypes (EMT) with the formation of filopodia and lamellipodia, and this was accompanied by EMT factors (increased vimentin and EMT-specific transcription factor slug, decreased E-cadherin) in all three CRC cell lines. We also found, with immunoblotting and immunofluorescence evaluation, clear TNF-β-induced expression of CSC-specific biomarkers (CD133, CD44, ALDH1) in all three CRC cells. These findings underline the essential impact of the tumor-promoting inflammation factor TNF-β as a dominant agent in promoting and supporting EMT and development of CSCs, thus, cancer progression and metastasis in CRC. In addition, these data are in accordance with findings from other studies, indicating that tumor cells change their morphology and phenotype from epithelial to higher cell motility and malignancy-mesenchymal phenotypes, and synthetize EMT-specific factors during tumorigenesis [23], accompanied by down-modulation of cell adhesion molecules (E-cadherin) and stimulation of vimentin, slug, and matrix metalloproteinase expression [55]. Furthermore, previous reports have demonstrated that the pro-inflammatory cytokine, TNF-α, is able to stimulate EMT in a various range of tumor cells [56]. Additionally, there have been greater amounts of TNF-α mRNA found in CRC cells, compared to normal epithelial colon cells [57]. Interestingly, it has been shown that EMT is an adequate triggering signal for CSC development and activation in malignancy and metastasis of cancer cells [25]. These data are in further accordance with previous studies, which have reported that EMT is crucial for tumor cell motility, migration, metastasis, and the CSC phenotype. Thus, epithelial tumor cells dissociate from adjacent cells and invade the surrounding tissue [58,59,60].

In correlation with the consideration that TNF-β as an inflammatory cytokine is a key decisive factor in cancer promotion, we showed that resveratrol, like anti-TNF-βR with TNF-β co-treatment, significantly decreased cell migration and EMT of CRC cells and promoted biochemical changes to MET with a planar cell surface and suppressed formation of CSC, and this was accompanied by a marked increase in apoptosis. Moreover, to show the anti-cancer impacts of resveratrol and anti-TNF-βR, we performed MTT and migration assays to determine the optimal suppression rate and cytotoxicity in all three CRC cell lines. In addition, pro-inflammatory cytokines have been noted to be synthesized through activated inflammatory cells, like macrophages in the tumor microenvironment [61], and during this process, the master pro-inflammatory transcription factor, NF-κΒ, is a major downstream target of cytokines, and NF-κΒ is a dominant agent for inducing and activating many tumor cells [62]. Furthermore, previous studies reported that the NF-κΒ signaling pathway was critical to cytokine-promoted migration and EMT in cancer cells [50,63]. Here, we showed that NF-κB is a crucial downstream target of TNF-β signaling and that it is a transcription regulator of TNF-β-induced FAK activation. Therefore, we used agents that block TNF-β-promoted NF-κΒ signaling, such as resveratrol. We have shown that resveratrol down-modulated TNF-β-activated NF-κB and NF-κB-promoted proteins, which are involved in invasion (MMPs), metastasis (CXCR4), and proliferation (Ki-67) in CRC cell lines. Indeed, these results are also consistent with those that have shown that resveratrol is a potent multitargeting anti-tumor agent and this effect is mediated partially through inhibiting NF-κB and NF-κB-promoted proteins in different tumor cells [13,30,54,64]. Interestingly, it has been shown previously, that the subcellular main target Sirt-1 protein for resveratrol is associated with NF-κB, and that Sirt-1 is able to deacetylate NF-κB on lysine 310, thus modulating NF-κB transcriptional activity [54,65].

We also showed that resveratrol induced nucleus pyknosis, chromatin condensation, and apoptotic body generation, indicating that resveratrol activated caspase-dependent cell death in CRC cells, which is in correlation with previous studies [32,54], highlighting how the resveratrol signaling pathway acts as an anti-tumor agent in CRC cells in a cytokine-promoted microenvironment.

It has been reported, that TNF-α as an activator and modulator of tumorigenesis induces the invasion abilities of tumor cells [61,66], but very little data is available regarding the impacts of TNF-β/TNF-βR on promoting tumor cell migration and EMT. In the present report, we demonstrated by immunoprecipitation assay that the impact of TNF-β-promoted CRC cell invasion and EMT points to a functional connection between TNF-βR and tumor-inducing biomarkers, such as FAK and NF-κB, suggesting that TNF-β increased the expression of TNF-βR, leading to FAK and NF-κB activation. When the TNF-βR pathway was suppressed by its specific antibody, the TNF-β/TNF-βR pathway appeared to lose its supporting role in CRC cell proliferation and migration. Our findings show that FAK is an intracellular molecular target of TNF-βR and, thus, tumor cell invasion. Indeed, it has been previously shown that FAK is significantly up-regulated in many cancer cells [30,67], involved in tumor cell survival, proliferation, and invasion [68], and that resveratrol clearly down-modulates cancer cell migration, viability, clonogenicity, and growth by suppression of FAK phosphorylation in CRC cells and other cancers [30,69,70,71]. The specific motility of cells is organized by polymerization and cross-linking of cytoskeleton proteins, like actin filaments conducting the formation of filopodia and lamellipodia, and they are stabilized through FAK during tumor cell migration [72,73]. Indeed, our findings provide insight into the determining role of TNF-β for EMT and tumor invasion in different CRC cells. These findings underline the significance of targeting TNF-β, and therefore, suppressing EMT and CSCs may be a promising approach to inhibiting the metastasis of tumor cells.

These data suggest for the first time the impact of TNF-βR on CRC cell EMT and migration, and underlines an important functional coherence between TNF-βR and cancer-stimulating factors, such as FAK, NF-κB, and NF-κB-related proteins.

## 5. Conclusions

**Figure 7:**
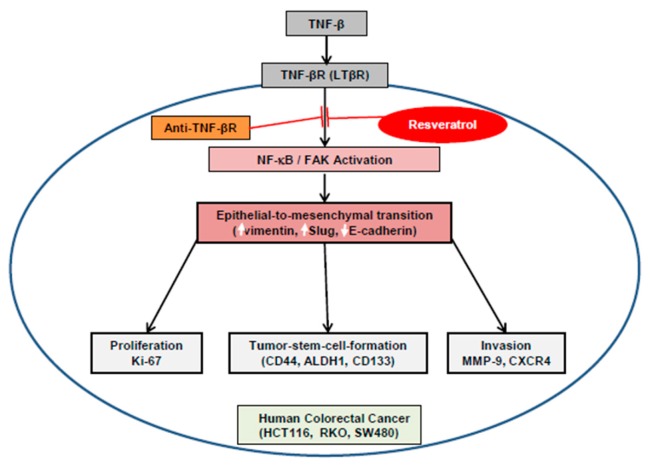
Graphic reveals resveratrol-modulated anti-cancer activity by suppressing TNF-β/TNF-βR in CRC cells.

This report examined the down-regulatory effects of resveratrol on TNF-β as a potent inflammatory cytokine, activating EMT in three CRC cell lines. Resveratrol, like anti-TNF-βR, significantly suppressed the TNF-β-induced EMT, EMT-associated factors, CSC formation, and migration of CRC cell lines. We found that TNF-β-promoted TNF-βR physically formed a protein complex with FAK and NF-κB and that these could be upregulated by TNF-β. More interestingly, this is the first study to indicate that FAK and NF-κB serve as a subcellular molecular downstream target of TNF-βR (Figure 7). Thus, our results present important evidence and data for future basic science investigations and clinical trials aiming to investigate the role of TNF-β/TNF-βR and resveratrol that will enable the development of a potential novel therapeutic strategy for CRC.
